# The Association of Ambient Air Pollution and Physical Inactivity in the United States

**DOI:** 10.1371/journal.pone.0090143

**Published:** 2014-03-05

**Authors:** Jennifer D. Roberts, Jameson D. Voss, Brandon Knight

**Affiliations:** 1 Department of Preventive Medicine and Biometrics, F. Edward Hebert School of Medicine, Uniformed Services University, Bethesda, Maryland, United States of America; 2 Epidemiology Consult Service, United States Air Force School of Aerospace Medicine, Wright-Patterson Air Force Base, Ohio, United States of America; University of Granada, Spain

## Abstract

**Background:**

Physical inactivity, ambient air pollution and obesity are modifiable risk factors for non-communicable diseases, with the first accounting for 10% of premature deaths worldwide. Although community level interventions may target each simultaneously, research on the relationship between these risk factors is lacking.

**Objectives:**

After comparing spatial interpolation methods to determine the best predictor for particulate matter (PM_2.5_; PM_10_) and ozone (O_3_) exposures throughout the U.S., we evaluated the cross-sectional association of ambient air pollution with leisure-time physical inactivity among adults.

**Methods:**

In this cross-sectional study, we assessed leisure-time physical inactivity using individual self-reported survey data from the Centers for Disease Control and Prevention's 2011 Behavioral Risk Factor Surveillance System. These data were combined with county-level U.S. Environmental Protection Agency air pollution exposure estimates using two interpolation methods (Inverse Distance Weighting and Empirical Bayesian Kriging). Finally, we evaluated whether those exposed to higher levels of air pollution were less active by performing logistic regression, adjusting for demographic and behavioral risk factors, and after stratifying by body weight category.

**Results:**

With Empirical Bayesian Kriging air pollution values, we estimated a statistically significant 16–35% relative increase in the odds of leisure-time physical inactivity per exposure class increase of PM_2.5_ in the fully adjusted model across the normal weight respondents (*p*-value<0.0001). Evidence suggested a relationship between the increasing dose of PM_2.5_ exposure and the increasing odds of physical inactivity.

**Conclusions:**

In a nationally representative, cross-sectional sample, increased community level air pollution is associated with reduced leisure-time physical activity particularly among the normal weight. Although our design precludes a causal inference, these results provide additional evidence that air pollution should be investigated as an environmental determinant of inactivity.

## Introduction

Worldwide, physical inactivity accounts for more than three million annual deaths and 6–10% of major non-communicable diseases, such as coronary heart disease, type-II diabetes and breast and colorectal cancers [1]–[5]. Similarly, physical inactivity is strongly associated with obesity and a portion of physical inactivity related mortality is attributed to obesity [6]–[9]. In the U.S., two-thirds of adults are overweight or obese and approximately six percent are extremely obese, which is a body mass index greater than or equal to 40.0 kg/m^2^ [10], [11]. While a majority of Americans are overweight or obese, sub-populations are disproportionately impacted. For instance, there are racial, ethnic, geographic and economic disparities in the obesity prevalence throughout the U.S. [12], [13]. Research into how the built environment may impact these disparities has shown conflicting results. [14], [15]. One explanation is that individual determinants interact with one another in a dynamic system, which suggests future research needs to account for the way factors interrelate with one another in the real world by using an ecological perspective.

Granted, modifiable lifestyle factors such as the increased consumption of unhealthy foods and physical inactivity are important independent contributors to the increasing burden of non-communicable disease. Other insidious factors, however, such as poor air quality, may influence physical inactivity, but current research has not adequately established this role. While not yet considered an environmental determinant of inactivity, there is little confusion about the unfavorable effects of acute and chronic air pollution exposure, particularly from particulate matter (PM) and ozone (O_3_), on both the respiratory and cardiovascular systems [16]–[19]. While some harms likely remain uncharacterized, research has shown that exposure to PM_2.5_ (particulate matter <2.5 µm in aerodynamic diameter), PM_10_ (particulate matter <10 µm in aerodynamic diameter) and O_3_ is associated with reduced exercise capacity, higher resting blood pressure, lower ventilator function and decrements in exercise performance [20]–[23]. Although there is abundant research illustrating these effects in a resting, inactive state, among athletes or normal weight subjects, the data examining the effects of poor air quality in real world settings are meager. Thus, the generalizability of these findings is in question particularly when over 60% of the U.S. population is overweight.

Another important gap is the difficulty in determining the geographic pattern of air pollution exposure. Although the U.S. Environmental Protection Agency (U.S. EPA) monitors and reports air pollution levels throughout the U.S., it is challenging to appreciate how these readings translate to air pollution exposures across standard geographic units, such as U.S. counties.

Thus, the overall aim of this study was to assess the association between ambient air pollution and leisure-time physical activity. Additionally, this association was examined after stratifying by body weight category.

## Materials and Methods

In this cross-sectional study, our two sources of data consisted of (1) annual summary measurements of 2011 ambient air quality monitoring data from the U.S. EPA Air Quality System (AQS) Data Mart and (2) Behavioral Risk Factor Surveillance System (BRFSS) survey data collected in 2011 throughout the U.S. that provided self-reported levels of leisure-time physical activity, demographic information and residential location. Using these data sources, we compared spatial interpolation methods to determine the best predictor for county-level PM_2.5_, PM_10_ and O_3_ exposures throughout the U.S and we evaluated the possible cross-sectional relationship of ambient air pollution with physical inactivity in the full study population and after stratifying by body weight.

### Physical Inactivity

BRFSS, a state-based telephone health survey system, collects data on behavioral and other health risk factors. As a whole, BRFSS, uses a methodology to collect a representative sample of the U.S. non-institutionalized adult population. As guided by the Centers for Disease Control and Prevention (CDC), data are collected from all 50 States, District of Columbia, Puerto Rico, U.S. Virgin Islands, and Guam. Although the CDC and other researchers have described the complex survey design in great detail, it should be noted that for many states, BRFSS is implemented through the use of disproportionate stratified random sampling (DSS) [24]–[26]. In order to account for the relatively recent rise in the proportion of U.S. households without landline telephones, adjustments were actualized during the fielding of the recent 2011 BRFSS to include households that rely on cellular telephones. Additionally, a more sophisticated weighting methodology known as “raking” was implemented. Raking, in contrast to the previously used poststratification method, forms individual variable adjustments in a series of data processing iterations, and thus reduces the risk of potential bias [27], [28].

Using BRFSS 2011 data, we assessed leisure-time physical inactivity through responses to the question, “During the past month, other than your regular job, did you participate in any physical activities or exercises such as running, calisthenics, golf, or walking for exercise?” The responses to this question were either “yes”, “no”, “don't know/not sure”, and “refused”. A response of “no” was defined as leisure-time physical inactivity.

In this study, inclusion criteria for the BRFSS data were as follows: (1) geographically located within the contiguous U.S including the District of Columbia; (2) responses from respondents who either were categorized as normal weight, overweight or obese; and (3) respondents from counties with both county and state Federal Information Processing Standard (FIPS) codes. Missing, “refused” and “don't know” responses were also excluded from the analysis.

### Air Pollution Exposure

The air quality data collected by the U.S. EPA AQS contains air monitoring measurements for criteria air pollutants from 1957 to present [29]. The database contains several million observations from thousands of monitors throughout the U.S. [29]. In addition to descriptive and geographic information about the monitoring sites, quality assurance information is also available.

For PM_2.5_ and PM_10_, annual summaries for 2011 were obtained using the standard 1-hour or 24-hour collection periods. Due to the strong seasonal and diurnal patterns that exist for ground level O_3_, U.S. EPA requires that monitoring locations collect only during specified months of the year as determined by their geographic location. Thus, the National Ambient Air Quality Standard (NAAQS) for ozone is based on an 8-hour averaging time. For inclusion in this study, the 2011 annual O_3_ summaries calculated using daily maximum 8-hour averages over the effective monitoring season were selected.

To confirm geographical accuracy, the monitoring data were mapped along with 2011 U.S. Census counties. County FIPS codes from the monitoring data were compared to county FIPS from the enclosing Census county. Of the 3945 records, four monitoring locations were found not to have a FIPS match. The spatial locations of these four were examined visually and the discrepant cases were located less than 500 meters from the county border, suggesting the discrepancy was due to error introduced during the import and processing of the air pollution data. Thus, no records were excluded from further analysis based on locational accuracy.

In order to ensure the accuracy and reliability of the air pollution concentrations, U.S. EPA inclusion criteria were applied. The inclusion criteria for the AQS data were as follows: (1) for PM_2.5_ and PM_10_: availability of greater than 75% of observations was required; (2) for O_3_: availability of greater than 75% of valid days in the effective monitoring season was required [29].

### Air Pollution Modeling

Since BRFSS data are available at the U.S. county level, the air pollution data provided from the discrete monitoring stations were modeled to estimate county-level average exposures. Studies examining the relationship between air pollution and health outcomes have implemented a variety of techniques to estimate pollution from U.S. EPA monitoring data, including various interpolation and spatiotemporal regression models [30]–[32]. With the use of ArcGIS 10.1, Inverse Distance Weighting (IDW) and Empirical Bayesian Kriging (EBK) were employed to perform spatial interpolation, creating continuous surfaces for the three air pollution parameters, which were then compared in order to select the best method for inclusion in subsequent analysis. A search window of 250 km was selected in order to ensure that air pollution estimates were generated for a substantial percentage of U.S. counties in the study area, while also maximizing the prediction precision.

The first method, IDW, is a deterministic method that imposes a model of spatial autocorrelation and calculates interpolation weights for each known point (in this case, each monitoring station) as a function of distance between the known points and the predicted points within a specific search window. There is an inverse relationship between the interpolation weights and the distance from the interpolated points to each known point. Hence, the values that are closer to the prediction location have more weight or influence on the predicted values than those farther away. We chose to calculate weights that change linearly in order to create a smooth surface. The second method, EBK, is an implementation of the kriging class of geostatistical methods that allow the development of a statistical autocorrelation model using a sample data set. Common kriging methods such as ordinary, simple, and universal kriging, require selection of model parameters from an empirical variogram. The empirical variogram is used to calculate interpolating weights such that the mean square error is minimized. EBK automates the parameter selection process through simulation and subsetting and generates accurate results from moderately non-stationary data, indicating that the mean and variance do not differ with geographical position [33], [34].

A one square kilometer grid was overlaid on a map of the counties that comprise the contiguous U.S. Each interpolation method produced air pollution estimates at the center of each square of the grid. County exposure estimates were calculated by averaging the interpolated values spatially located within the county borders. U.S. counties that did not have a single interpolated estimate within its borders were excluded from further analysis.

Cross-validation was completed for each interpolation method and for each pollutant to test the generalization performance and provide a quantitative comparison of the IDW and EBK methods. This was performed by omitting values for a single monitor and then using the remaining monitors to interpolate the concentration at the removed monitor's location. To identify and select the most appropriate interpolation method to use for further analysis, the cross-validations were compared in terms of prediction root mean square error (RMSE) and prediction mean absolute error (MAE).

Upon this selection, the annual mean of PM_2.5_, PM_10_ and O_3_ concentrations were transformed from continuous variables to categorical variables using natural breaks classification method with Jenks optimization. Natural breaks are data-specific classes, which are based on natural groupings inherent in the data and where boundaries are set based on relatively large differences in the data values by reducing the within and maximizing the between class variance. Finally, we linked annual average concentrations of PM_2.5_, PM_10_ and O_3_ with the 2011 BRFSS data using FIPS codes as the linking unit.

### Statistical Analysis

BRFSS uses a complex survey design with stratification, multistage clustering and sampling weights. Therefore, statistical analysis was performed in STATA MP/12.1 using the -svyset- commands. The weighted prevalence of leisure-time physical inactivity was calculated by physical demographic and risk factor categories. Additionally, the weighted prevalence of demographic and other risk factor variables were calculated by air pollution exposure class. Chi-square tests for homogeneity were performed to investigate the association of the demographic and behavioral risk variables with physical inactivity and air pollution exposure.

We considered whether adults who were exposed to higher levels of PM_2.5_, PM_10_ and O_3_ concentrations exhibited higher levels of physical inactivity by performing logistic regression. Additionally, we examined this association after stratifying the data into three subgroups [e.g. (1) normal weight (body mass index (BMI) 18.5 to 24.9); (2) overweight (BMI 25.0 to 29.9), and (3) obese (BMI 30 or higher)] as defined and categorized by the BRFSS data. The air pollution variables were analyzed both as continuous and natural breaks categorical variables using three models for each pollutant. Model A examined the effect on physical inactivity with ambient exposure to PM_2.5_, PM_10_ and O_3_ without the adjustment of any confounders. Model B adjusted for age, sex, race/ethnicity, education, annual income, marital status, seasonality and geographic region. Along with the aforementioned confounders, Model C, also adjusted for general health status, smoking, disability, asthma, urbanization and the other two air pollutants. Additionally, we calculated Pearson's correlation coefficients to examine relations between the three pollutant measures.

### Ethics Statement

The Uniformed Services University of the Health Sciences, Human Research Protections Program Office, determined that this research was non-human subjects research consistent with 32 CFR 219.102.

## Results

The 2011 BRFSS dataset encompassed 504,408 observations. Based on the inclusion and exclusion criteria, data from 48 states and the District of Columbia were included in the analysis (N = 329,628 subjects from 2249 U.S. counties). A total of 24.5% (n = 80,825) responded “no” to participating in any physical activity during the previous month. The prevalence of leisure-time physical inactivity was higher among older respondents and among females; however with respect to race and ethnicity, Black/non-Hispanic respondents demonstrated the highest prevalence of physical inactivity, which was closely followed by Hispanic respondents. The highest levels of reported physical inactivity were also observed among the respondents with lower levels of education, those who reported being divorced, widowed or separated, and, those receiving less than $50,000 in annual income. Respondents who reported obesity, disability, asthma, or prior history of smoking were also more likely to report physical inactivity. Physical inactivity was highest during the months of January through March and October through December. In addition, physical inactivity was highest in the Southeastern part of the U.S. while also being lowest in the West. There was also a much higher level of physical inactivity among respondents who resided in rural counties or non-metropolitan counties (Data not shown, *p*-value <0.001).

The two interpolation methods, IDW and EBK, were evaluated based on accuracy and precision for predicting annual ambient PM_2.5_, PM_10_ and O_3_ concentrations. Results showed that the IDW and EBK models were similar in regards to RMSE and MAE with EBK trending toward increased precision for all three air quality parameters. Thus, EBK was identified as the most appropriate method. Furthermore, kriging interpolation methods have been recognized by U.S. EPA as possessing the greatest merit in predicting air pollution concentrations in unknown locations [35].

**Figure 1:**
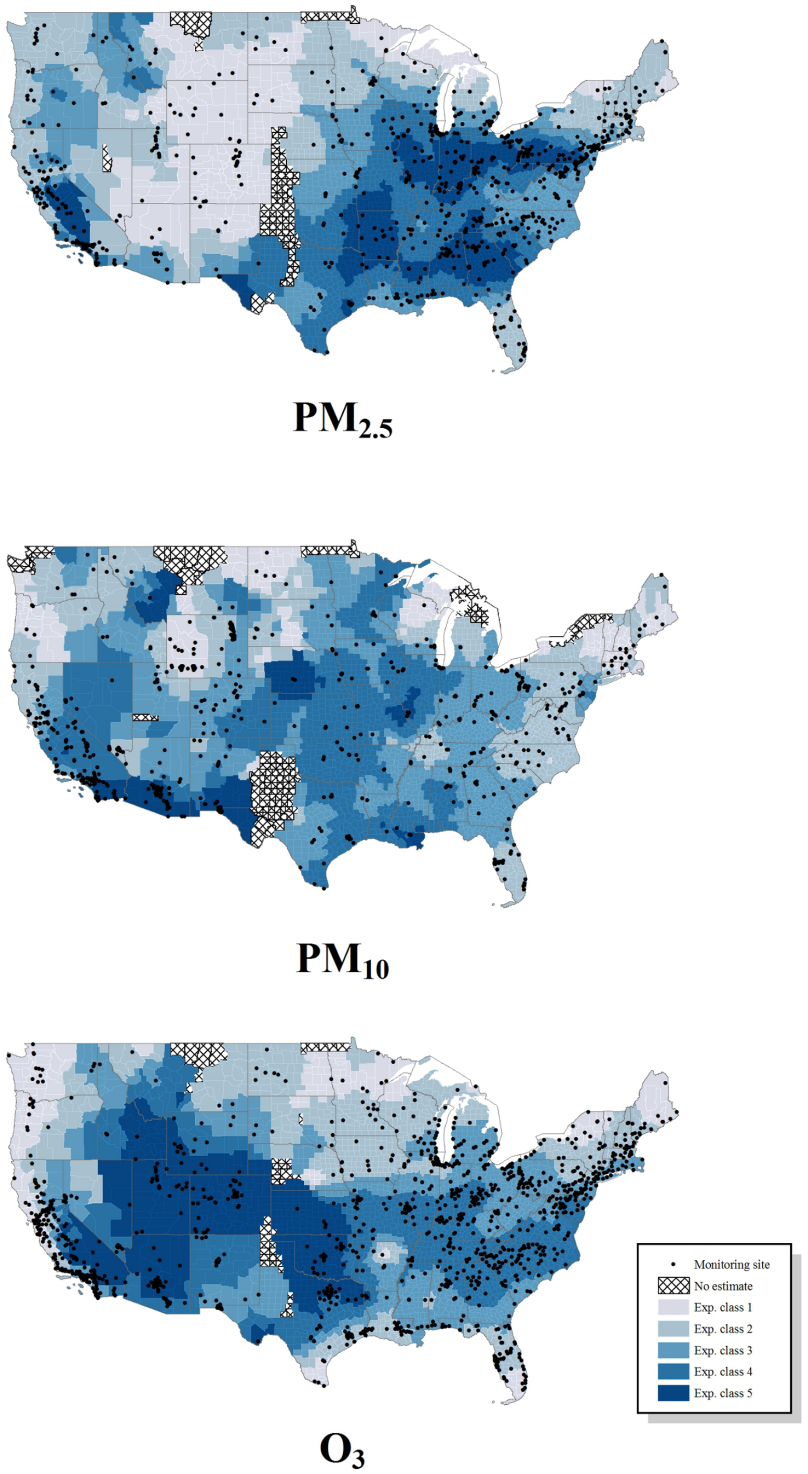
U.S. map of annual mean Empirical Bayesian Kriging (EBK) interpolated ambient air pollution concentrations by natural breaks classes.

The mean of PM_2.5_, PM_10_ and O_3_ annual mean concentrations were 9.50 ug/m^3^ [SD: 1.80], 19.52 ug/m^3^ [SD: 4.16], and 45 ppb [SD: 4.48] for the BRFSS counties, respectively. All of the pollutants were positively correlated with PM_2.5_ and PM_10_ having the strongest correlation (r: 0.29) followed by O_3_ and PM_10_ (r: 0.23). Furthermore, the air pollution variables were transformed into categorical variables using the Jenks' natural breaks methodology (Table 1). While the highest concentrations of PM_2.5_ were found in the upper Atlantic, Midwest, and the South, along with a small cluster in Southern California, the higher concentration PM_10_ counties were clustered throughout the U.S., particularly in the Southwest (Figure 1). The highest two classes of O_3_ were clustered in the middle and western part of the country (Figure 1). When comparing air pollution by natural breaks class, the most evident differences were observed within the race/ethnicity, U.S geographic region and metropolitan county classification categories among all of the air pollutants (Tables 2–3). Generally, White/non-Hispanic respondents and those living in rural counties or with a population of less than 250,000 were exposed to lower levels of PM_2.5_ and PM_10_ (Tables 2–3).

**Table 1:** Annual means of PM_2.5_, PM_10_ and O_3_ Empirical Bayesian Kriging (EBK) interpolated ambient air pollution concentrations by natural breaks classes.

Air Pollution Classes	PM_2.5_ (ug/m^3^)	PM_10_ (ug/m^3^)	O_3_ (ppb)
Class 1	3.49–6.52	5.00–13.40	26.93–37.83
Class 2	6.53–8.45	13.41–17.59	37.84–42.40
Class 3	8.46–9.85	17.60–21.27	42.41–45.83
Class 4	9.86–10.89	21.28–26.31	45.84–49.89
Class 5	10.90–15.38	26.32–52.88	49.90–56.94

**Table 2:** Prevalence of demographic factors by air pollution exposure class.

Demographic Factors	Prevalence (%)^a^					
	PM_2.5_ b		PM_10_ b		O_3_ b	
	Class 1 (n = 32,438)	Class 5 (n = 57,600)	Class 1 (n = 42,539)	Class 5 (n = 16,081)	Class 1 (n = 31,333)	Class 5 (n = 45,901)
**Age** *						
18–24 years	9.07	10.71	10.07	11.07	9.77	11.29
25–34 years	17.04	17.55	15.06	18.46	16.62	18.92
35–44 years	19.05	19.13	17.13	19.31	19.13	19.54
45–54 years	20.91	20.36	21.80	19.20	20.47	19.92
55–64 years	17.48	15.87	17.79	15.20	16.52	15.04
≥65 years	16.45	16.38	18.16	16.76	17.49	15.29
**Sex**						
Male	51.71	51.15	51.84	50.12	51.28	51.32
Female	48.29	48.85	48.16	49.88	48.72	48.68
**Race/Ethnicity** *						
White/non-Hispanic	75.81	64.28	85.48	59.33	62.54	65.18
Black/non-Hispanic	2.93	13.15	3.68	9.56	5.41	7.24
Hispanic	13.62	15.90	5.38	24.29	19.92	20.64
Asian/Pacific Islander	2.52	4.29	2.44	4.25	8.80	3.20
American Indian	3.56	0.87	1.12	0.98	1.19	2.18
Multi/Other	1.56	1.52	1.90	1.60	2.14	1.56
**Educational Level** *						
Not graduated from high school	10.49	15.19	9.19	15.50	12.02	14.58
High school graduate	27.25	29.59	28.06	25.45	23.85	25.75
Attended college	33.57	29.64	31.66	33.72	32.81	34.26
College graduate or higher	28.70	25.58	31.09	25.33	31.32	25.42
**Annual Income Level** *						
<$25,000	28.27	32.44	25.21	31.80	28.63	29.91
≥$25,000 to <$50,000	26.84	25.14	25.24	25.79	24.47	26.28
≥$50,000	44.89	42.42	49.55	42.41	46.90	43.81
**Marital Status** *						
Married/partnered	61.09	57.44	59.35	56.30	58.53	60.55
Divorced/widowed/separated	20.15	19.62	19.24	20.86	19.52	19.27
Never married	18.76	22.93	21.41	22.84	21.95	20.18

a Proportions based on frequency-weighted final weight variable rounded to nearest integer.

b Air pollution based on five natural breaks air pollution classes (Classes 2-4 not shown).

*p-value <0.0001 Chi-square test of homogeneity – All three pollutants.

**Table 3:** Prevalence of risk or geographic factors by air pollution exposure class.

Risk/Geographic Factors	Prevalence (%)^a^					
	PM_2.5_ b		PM_10_ b		O_3_ b	
	Class 1 (n = 32,438)	Class 5 (n = 57,600)	Class 1 (n = 42,539)	Class 5 (n = 16,081)	Class 1 (n = 31,333)	Class 5 (n = 45,901)
**Smoking** *						
Current and former smoker	48.24	44.80	48.27	44.08	43.39	42.92
Never smoked	51.76	55.20	51.73	55.92	56.61	57.08
**Body Mass Index** §						
Normal weight	37.13	33.41	35.63	34.65	36.51	35.37
Overweight	37.58	36.93	37.72	37.27	37.41	36.83
Obese	25.29	29.65	26.65	28.08	26.08	27.81
**Disability** *						
No	76.32	77.11	75.24	76.57	74.98	77.00
Yes	23.68	22.89	24.76	23.43	25.02	23.00
**General Health Status** *						
Excellent/good	84.69	81.50	85.38	81.94	82.93	83.52
Fair/poor	15.31	18.50	14.62	18.06	17.07	16.48
**Asthma Currently** ¶						
No	91.09	91.72	89.88	91.80	91.03	91.51
Yes	8.91	8.28	10.12	8.20	8.97	8.49
**Seasonality** *						
Quarter 1 (January to March)	17.13	23.89	27.70	25.40	27.27	20.62
Quarter 2 (April to June)	27.36	23.40	26.88	27.16	25.48	29.27
Quarter 3 (July to September)	30.13	26.86	23.71	21.94	23.80	24.91
Quarter 4 (October to December)	25.39	25.86	21.71	25.49	23.45	25.20
**U.S. Geographic Region** *						
Northeast	6.79	10.89	68.89	0.00	7.82	0.00
Southeast	0.00	20.73	0.58	1.26	19.51	0.00
Midwest	11.04	33.58	5.59	19.47	4.02	4.04
Southwest	56.50	12.90	1.61	16.26	5.58	59.12
West	25.67	21.89	23.53	62.74	63.07	36.84
**Metropolitan County Classification** *						
Rural counties	29.84	14.03	21.43	5.37	14.17	12.67
Counties with <250,0000	21.87	9.14	10.12	7.61	8.04	8.49
Counties with 250,000–1 Million	14.86	21.19	23.40	12.59	20.00	20.94
Counties with ≥1 Million	33.43	55.63	45.06	74.44	57.79	57.90

a Proportions based on frequency-weighted final weight variable rounded to nearest integer.

b Air pollution based on five natural breaks air pollution classes (Classes 2–4 not shown).

*p-value <0.0001 Chi-square test of homogeneity – All three pollutants.

§ *p*-value <0.0001 Chi-square test of homogeneity – Only PM_2.5_ and O_3_.

¶ *p*-value <0.0001 Chi-square test of homogeneity – Only PM_10_ and O_3_.

### Association of Physical Inactivity and Air Pollution Exposure by Body Weight

When considering PM_2.5_ as a continuous variable, the odds of leisure-time physical inactivity significantly increased with increasing concentration of PM_2.5_ across all models and strata. For the fully adjusted Model C, we estimated a 2.4% relative increase in the odds of physical inactivity per µg/m^3^ increase of PM_2.5_ exposure among the obese respondents [OR = 1.02 (95% CI: 1.00, 1.05)]. Similarly, increasing concentration of PM_10_ among the normal weight respondents was also associated with higher odds of inactivity [OR = 1.01 (95% CI: 1.00, 1.02)]. We also estimated increases in the odds of physical inactivity per unit increase of each air pollutant for the entire combined dataset and those results were also found to be statistically significant for PM_2.5_ in all models (Data not shown, *p*-value <0.01).

**Figure 2:**
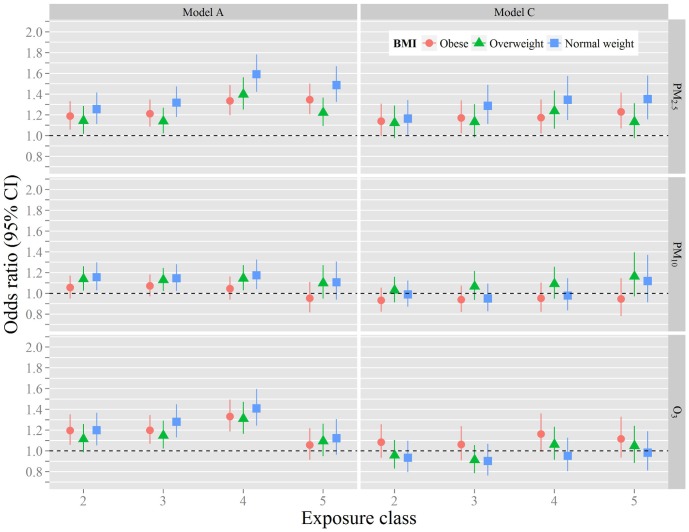
Association between air pollution exposure classes and the odds ratios of physical inactivity ^†, ††, †††^. † Model A unadjusted. †† Model C adjusted for age, sex, race/ethnicity, education, annual income, marital status, seasonality, geographic region, general health status, smoking, disability, asthma, urbanization, and the other two air pollutants. ††† Exposure class 1 is referent.

Alternatively, the associations with inactivity were also modeled using natural breaks exposure classes for each of the three pollutants (PM_2.5_, PM_10_ and O_3_). There was a statistically significant 16–35% relative increase in the odds of physical inactivity per increase from the lowest PM_2.5_ exposure class in the fully adjusted Model C among the normal weight respondents (Table 4), which was a stronger association than the other body weight strata. Figure 2 illustrates a relationship between the odds of physical inactivity and increasing dose as represented by exposure class. For O_3_, statistical significance was observed only in the unadjusted Model A for all three weight groups (Figure 2). Results found using the full dataset were similar to that of the normal weight stratum (Data not shown).

**Table 4:** Association between Empirical Bayesian Kriging (EBK) interpolated ambient PM_2.5_ exposure class and physical inactivity by body weight subset, logistic regression model.

PM_2.5_ Exposure Class	Obese Weight (n = 99,699)			Overweight (n = 127,720)			Normal Weight (n = 116,927)		
	Logistic Regression Models*								
	A^a^	B^b^	C^c^	A	B	C	A	B	C
	OR (95% CI)			OR (95% CI)			OR (95% CI)		
Class 1	Referent	Referent	Referent	Referent	Referent	Referent	Referent	Referent	Referent
Class 2	1.19 (1.06, 1.33)	1.11 (0.98, 1.25)	1.14 (0.99, 1.31)	1.14 (1.02, 1.28)	1.10 (0.97, 1.25)	1.12 (0.98, 1.29)	1.26 (1.11, 1.42)	1.16 (1.02, 1.32)	1.16 (1.01, 1.34)
Class 3	1.21 (1.09, 1.35)	1.14 (1.02, 1.29)	1.17 (1.02, 1.34)	1.14 (1.02, 1.27)	1.13 (0.99, 1.28)	1.13 (0.98, 1.30)	1.32 (1.18, 1.47)	1.24 (1.09, 1.40)	1.29 (1.11, 1.49)
Class 4	1.33 (1.20, 1.49)	1.15 (1.02, 1.30)	1.17 (1.02, 1.35)	1.40 (1.25, 1.56)	1.24 (1.09, 1.40)	1.24 (1.07, 1.43)	1.59 (1.42, 1.78)	1.30 (1.14, 1.48)	1.35 (1.16, 1.58)
Class 5	1.35 (1.21, 1.50)	1.21 (1.07, 1.36)	1.23 (1.07, 1.42)	1.22 (1.09, 1.37)	1.13 (0.99, 1.28)	1.13 (0.98, 1.31)	1.49 (1.33, 1.67)	1.27 (1.12, 1.44)	1.35 (1.16, 1.58)

a Model A unadjusted.

b Model B adjusted for age, sex, race/ethnicity, education, annual income, marital status, seasonality and geographic region.

c Model C adjusted for age, sex, race/ethnicity, education, annual income, marital status, seasonality, geographic region, general health status, smoking, disability, asthma, urbanization, and the other two air pollutants.

*Wald Chi-square *p*-value <0.0001 for all three models.

Lastly, the relationship between air pollution and several other covariates was notable. For instance, odds ratios for physical inactivity increased strongly with increasing age and BMI classes (Table 5). By contrast, higher levels of education and income classes decreased the odds of physical inactivity. Furthermore, respondents in the Western part of the U.S had 35% higher odds of being active than those in the Northeast (Table 6). There was also a 32–33% higher odds of activity found among respondents during the warmer months of the year (Table 6). Finally, the odds of physical inactivity decreased with increasing urbanization (Table 6).

**Table 5:** Association of demographic factors with physical inactivity.

Demographic Factors	OR (95% CI)
**Age** *	
18–24 years	Referent
25–34 years	1.51 (1.36, 1.67)
35–44 years	1.72 (1.55,1.91)
45–54 years	1.74 (1.57, 1.92)
55–64 years	1.82 (1.64, 2.01)
≥65 years	2.00 (1.80, 2.21)
**Sex** *	
Male	Referent
Female	1.15 (1.10, 1.19)
**Race/Ethnicity** *	
White/non-Hispanic	Referent
Black/non-Hispanic	1.19 (1.13, 1.27)
Hispanic	1.22 (1.14, 1.30)
Asian/Pacific Islander	1.80 (1.58.2.05)
American Indian	0.92 (0.79, 1.07)
Multi/Other	0.91 (0.80, 1.03)
**Educational Level** *	
Not graduated from high school	Referent
High school graduate	0.84 (0.79, 0.90)
Attended college	0.62 (0.58, 0.66)
College graduate or higher	0.42 (0.39, 0.45)
**Annual Income Level** *	
<$25,000	Referent
≥$25,000 to <$50,000	0.96 (0.91, 1.00)
≥$50,000	0.72 (0.69, 0.76)
**Marital Status**	
Married/partnered	Referent
Divorced/widowed/separated	1.02 (0.98, 1.06)
Never married	0.97 (0.92, 1.03)

Adjusted for all above variables in the table and all three air pollution variables.

*Joint adjusted Wald test *p*-value <0.0001.

**Table 6:** Association of risk or geographic factors with physical inactivity.

Risk/Geographic Factors	OR (95% CI)
**Smoking**	
Never smoked*	Referent
Current and former smoker	1.18 (1.14, 1.22)
**Body Mass Index** *	
Normal weight	Referent
Overweight	1.04 (1.00,1.09)
Obese	1.44 (1.38, 1.50)
**Disability** *	
No	Referent
Yes	1.42 (1.37, 1.48)
**General Health Status** *	
Excellent/good	Referent
Fair/poor	1.74 (1.66, 1.82)
**Asthma Currently**	
No	Referent
Yes	0.98 (0.92, 1.03)
**Seasonality** *	
Quarter 1 (January to March)	Referent
Quarter 2 (April to June)	0.68 (0.65, 0.72)
Quarter 3 (July to September)	0.67 (0.63, 0.70)
Quarter 4 (October to December)	0.84 (0.80, 0.88)
**U.S. Geographic Region** *	
Northeast	Referent
Southeast	1.01 (0.96, 1.06)
Midwest	0.90 (0.86, 0.95)
Southwest	0.89 (0.84, 0.96)
West	0.65 (0.61, 0.69)
**Metropolitan County Classification** *	
Rural counties	Referent
Counties with <250,0000	0.93 (0.89, 0.99)
Counties with 250,000–1 Million	0.90 (0.85, 0.94)
Counties with ≥1 Million	0.88 (0.84, 0.92)

Adjusted for all above variables in the table and all three air pollution variables.

*Joint adjusted Wald test *p*-value <0.0001.

## Discussion

In this nationally representative, cross-sectional sample, increased ambient levels of PM_2.5_, PM_10_ and O_3_ were associated with reduced physical activity. This association was significant in all models of adjustment for PM_2.5_. Our research demonstrated an association between increasing ordinal air pollution classes and increasing odds of inactivity among adults. Remarkably, the most compelling relationships were evident among normal weight as opposed to overweight or obese respondents. These findings suggest that the presence of air pollution may discourage normal weight individuals from engaging in leisure-time physical activity.

Our research exhibited an inverse relationship between air pollution exposure and leisure-time physical activity among Americans. This is consistent with findings from similar studies that also examined the association of physical inactivity and air pollution and found a direct relationship with increasing PM_2.5_ and the increasing prevalence of physical inactivity (*p*-value <0.01) [36]. This prior work, however, only examined crude levels of PM_2.5_ and generalized findings across all weight categories. Another study modeled other air pollutants, including O_3_ and nitrogen oxides (NO_x_), and physical inactivity limited to Southern California, but did not consider air pollution as a determinant of inactivity [37].

We add to this literature by using more recent data, sophisticated interpolation methods increasing the coverage and modeling reliability, and clarifying the association across weight strata. Our research revealed a higher prevalence of physical inactivity among obese and overweight, as compared to normal weight, respondents. When stratifying by body weight category, the association between air pollution and leisure-time physical activity varied minimally by body weight category with the exception of PM_2.5_ where the magnitude of association trended higher among the normal weight respondents. One reason for this finding may be due to the fact that normal or healthy weight adults already are more physically active than obese or overweight adults and therefore the reduction in activity is greater. For instance, obesity related disability may create a disparity in discretionary activity - a lean population is active when the conditions are favorable while those who are disabled are inactive regardless.

Although a causal mechanism cannot be elucidated by our study design, the association of physical inactivity and ambient air pollution as mediated by body weight category is plausible. Positive associations have been found with exposure to air pollutants and direct health effects on the respiratory and cardiovascular systems, such as increased blood pressure, asthma exacerbations, cardiac arrhythmia, and decreased lung function [17], [38]–[41]. Therefore, adverse health effects from increasing levels of air pollution could reduce one's capacity for physical activity. In addition to the physiological effects (e.g. difficulty breathing), the possibility of a psychosocial effect (e.g. smog appearance disincentivizing physical activity) could be contributing factor to this association. With readily available information, the U.S. population is likely more aware of the health risk associated with exposure to high levels of air pollution. Hence, this awareness may ultimately discourage individuals from engaging in outdoor physical activity.

While these are important findings, one study limitation was potential misclassification of exposure based on the air pollution modeling, interpolated estimates and annual means. Because we applied the same methods uniformly throughout the entire U.S. we suspect this biases to the null as non-differential measurement error, but we cannot rule out differential misclassification given the geographic variation in our exposure and outcome. Furthermore, the air pollution data used were at the county-level and did not provide information at an individual daily exposure level. Yet, since PM_2.5_ and O_3_ are often more homogeneous air pollutants in their distribution over large regions, we believe this misclassification was minimized.

Our study used self-reported leisure-time physical activity and self-reported data are often subject to certain biases. Since physical activity was assessed as a dichotomous variable over a month timeframe, the accuracy of responses may not have been compromised. However, the BRFSS physical activity question provided examples of exercise such as running and calisthenics. Physical activity includes not only the participation of sports and exercise, but also walking or biking on a daily basis by means of an active commute or transport. With the BRFSS examples, respondents may not have considered their less obvious physical activities, such as walking to work because the survey question asked respondents about participation in physical activities outside of their “regular job”. Additionally, other built environmental factors, such as neighborhood walkability or safety, may have influenced one's level of physical activity.

The major strength of this study is the use of our novel EBK estimations for air pollution exposures. Unlike other kriging methods, EBK allows for automated model fitting and more accurate and robust predictions. Another strength of this study is the use of a nationally representative sample with extensive demographic, behavioral and risk factor data. The combination of BRFSS data with U.S. EPA air pollution data brought to light novel findings. Lastly, we were able to examine the relationships of three air pollutants and their influence with each other on physical inactivity.

The findings of this research emphasized the phenomenon that there is a complex interplay among many risk factors, behavioral and demographic variables, which are associated with physical activity. Thus, the complexity limits the applications of observational research as it can raise questions of causality and directionality. Because PM_2.5_ is a modifiable exposure with cost effective mitigation strategies, future research could evaluate causality by cluster randomizing the timing of PM_2.5_ reduction interventions and assessing the short-term impact on leisure-time physical inactivity [42], [43].

## Conclusions

We present evidence that as air pollution concentrations increase, American adults, especially those who are lean, are less likely to be physically active. Given the public health emphasis on community level determinants of inactivity, additional research should determine if environmental air pollution is a modifiable risk factor for inactivity. We postulate those interventions which improve physical activity and reduce air pollution such as transportation interventions will have both primary and secondary benefits.
